# Thermal Imaging of Tongue Surface as a Prognostic Method in the Diagnosis of General Diseases—Preliminary Study

**DOI:** 10.3390/jcm12216860

**Published:** 2023-10-30

**Authors:** Daria Wziątek-Kuczmik, Iwona Niedzielska, Aleksandra Mrowiec, Agata Stanek, Piotr Gościniewicz, Ewa Mrukwa-Kominek, Armand Cholewka

**Affiliations:** 1 Department of Cranio-Maxillofacial Surgery, Faculty of Medical Sciences, Medical University of Silesia, 40-055 Katowice, Poland; iedzielska.konsultant@wp.pl; 2 Faculty of Science and Technology, University of Silesia, 40-007 Katowice, Poland; starostafm00@gmail.com (A.M.); armand.cholewka@gmail.com (A.C.); 3 Department and Clinic of Internal Medicine, Angiology and Physical Medicine, Faculty of Medical Sciences in Zabrze, Medical University of Silesia, Batorego 15 St, 41-902 Bytom, Poland; astanek@tlen.pl; 4 Department of Ophthalmology, Professor K. Gibiński University Clinical Center, Medical University of Silesia, 40-055 Katowice, Poland; piotr.gosciniewicz@gmail.com; 5 Department of Ophthalmology, Faculty of Medical Sciences in Katowice, Medical University of Silesia, 40-055 Katowice, Poland; emrowka@poczta.onet.pl

**Keywords:** tongue, medical diagnostic, dynamic infrared thermal image, tongue temperature, imaging method

## Abstract

The aim of this work was to develop an original method of thermographic measurement of tongue temperature as a prognostic method in the diagnosis of general diseases. This study examined the temperature distribution on the dorsal and apical surfaces of the tongue in patients with various general diseases and introduced a procedure for cooling the oral cavity. Patients with a high risk of systemic infections were referred to the Oral and Maxillofacial Surgery Outpatient Clinic of the Medical University of Silesia (SUM) in Katowice to identify potential foci of dental infections. They underwent an evaluation of their dentition as well as a prognostic tongue examination using the thermal camera FLIR T540 with a sensitivity of <0.03 K. The obtained results revealed statistically significant differences in the tongue average temperature parameters between the two study groups—healthy patients and patients with disorders. We obtained median temperatures for tongue dorsum (TD) of 35.8 °C and 35.2 °C for healthy patients and patients with disorders, respectively. Also, statistical differences have been obtained for tongue apex (TA) average temperatures. They were 35.5 °C and 34.0 °C for healthy and patients, respectively (*p* = 0.0001). Similar statistical results presented significant differences in the temperature difference (defined as DT = average temperature 10 min − average temperature 2 min after rinsing of the mouth) of the examined areas of the tongue between the healthy temperature and the unhealthy patient’s temperature. It seems that thermal imaging has potential in the prevention and diagnosis of general diseases.

## 1. Introduction

The stability of the internal temperature of the body is conditioned by maintaining a balance between the processes of heat loss and production. Thermal equilibrium is a dynamic process influenced by metabolic processes occurring in individual systems, as well as changing environmental conditions [1]. The relationship between human body temperature and disease dates back to the beginnings of medicine. For generations, doctors have made a diagnosis based on body temperature measurements with simple clinical thermometers. It was assumed that the value of the internal temperature is close to the value of the rectal temperature and is in the range of 36–38 °C, while the value of the temperature within the oral cavity and on the tympanic membrane is usually lower by about 0.7 °C. On the other hand, the temperature value in the armpit ranges from 36.28 °C to 37.58 °C and strongly correlates in infrared thermography with the temperature value of the outer corner of the eye [2]. Moreover, the temperature of the outer corner of the eye is prognostic in the fever screening method of non-invasive thermovision measurements in children, as well as in a large population of people in public spaces [1,2]. In recent years, there has been a dynamic development of applications in medicine of thermal imaging as a non-invasive repeatable method with high thermal sensitivity (reaching 0.03 °C) and accuracy of quantitative temperature assessment. Due to the great need to implement effective and rapid diagnostic screening tests, hopes are placed on the use of infrared thermography for these purposes. In the diagnosis of many diseases, in which the surface temperature of the skin may reflect the presence of inflammation in the underlying tissues, and where blood flow is increased or decreased due to clinical abnormalities or drug therapy, temperature assessment is crucial. It is also used as a diagnostic test and to assess the clinical results of treatment [1,3,4,5,6,7,8,9,10,11,12,13,14].

This work presents a pilot study using infrared thermography in tongue temperature measurement as a non-invasive, aseptic, harmless technique (without ionizing radiation), and, at the same time, ensures relatively low costs of the procedure. We undertook an attempt to assess the temperature of the tongues for the prognostic purposes of general diseases manifested by fluctuations in internal temperature.

It can be found in the literature that the tongue is a “health mirror” that reflects the patient’s health condition. It is also greatly available during a dental examination [9]. Therefore, the authors developed their method for assessing the tongue surface temperature for prognostic purposes of general diseases. This non-invasive, non-contact test can alert doctors to the diagnosis of concomitant disease. Dynamic changes in tongue temperature occur, for example, due to mouth cooling. They will be the subject of the conducted research. It may give quite clear correlations with a specific disease. In this way, at the very initial stage of dental treatment, you can contribute to taking the right steps and implementing appropriate medical procedures. The proof of such a phenomenon is the main goal of this work.

## 2. Materials and Methods

Patients of the Outpatient Clinic of Maxillofacial Surgery of the SUM in Katowice, directed to eliminate potential foci of infection belonging to the high-risk group of systemic infections, underwent a tongue temperature screening. Each patient underwent a clinical examination, following the prevailing algorithm of examinations for the assessment of foci of infection in the oral cavity.

A strongly muscled mobile organ resting freely in the bottom of the oral cavity is easily subjected to non-contact examination. In our work, we focused on the assessment of tongue temperature, as its value significantly correlates with blood perfusion rate and metabolic processes.

The tongue was divided into two sectors: the dorsum (TD) and the apex (AT) of tongue in order to describe the anomalies.

The inclusion criterion was adult patients of both sexes, diagnosed with a general disease and having a healthy tongue without pathological changes and of normal color.

The exclusion criterion was patients with elevated body temperature, with an active inflammatory process of bacterial or viral origin, or with a neoplastic process in the area of the head and neck. This study was approved by the Bioethics Committee of SUM Resolution No. PCN/CBN/0052/KB1/67/I/22.

This study was performed on 33 patients who were high-risk patients due to the diagnosis of the systemic disease. The study group consisted of individuals aged 22 to 68 years (15 women and 18 men) with an average BMI of approximately 27. The control group included 33 healthy volunteers aged between 23 and 63 years (17 women and 16 men), with an average BMI of around 24. In the research conducted, it was found that the average age within the study group was 40, whereas in the control group, it stood at 39.

There were ophthalmology, hematology, nephrology, and cardiology patients involved in the study group. The biggest subgroup of patients complained about inflammatory or optic nerve atrophy and inflammation of the iris and ciliary body. Moreover, there were patients with kidney graft, nephropathy, myeloma, Hodgkin’s lymphoma, and diabetes, which was observed in most patients.

### Infrared Thermography Measurements

The thermal imaging examination was carried out in strictly defined and repeatable conditions by a team of the same researchers. The patients were always tested in the same room, with a constant temperature of 21 + 0.5 °C and humidity between 50% and 60%, with no air conditioning, and with the windows and doors closed to eliminate the flow of air around the tested patient. Each patient underwent 20 min of acclimatization to the conditions in the room. Body temperature, blood pressure, pulse, and BMI were measured. Prescribed drug treatments were carefully recorded.

The examination was carried out on a dental chair, with the patient’s head supported on a headrest and with the chin in a plane parallel to the ground. After rinsing the mouth with water at room temperature for 60 s, they were asked to close their mouths for 10 min before the measurement, leaving the tongue still. Two test cycles were performed, after 2 and 10 min, with two complete measurement cycles according to the literature [9,10]. 

Thermal imaging was conducted just after the mouth was opened (0), and then the first test (I) was carried out after 2 min, with the mouth wide open, subjecting the tongue, lying freely on the floor of the mouth. And the second test (II) was performed analogously after another 8 min—the muscle was relaxed, and the apex of the tongue was directed slightly downwards.

The average temperature of selected sectors of the tongue surface was measured using FLIR T540 thermal camera, with a sensitivity of <0.03 K. Statistica 10 program was used to carry out the statistical analysis. Following proper statistical analysis, normality was verified using the Shapiro–Wilk test, and homogeneity of variance was assessed using Levene’s test. Subsequently, the Mann–Whitney U test was applied, as it was found that the study variables did not show a normal distribution, nor did they meet the assumption of homogeneity of variance. The differences were statistically significant at a level of significance of *p* < 0.05.

## 3. Results

**Figure 1:**
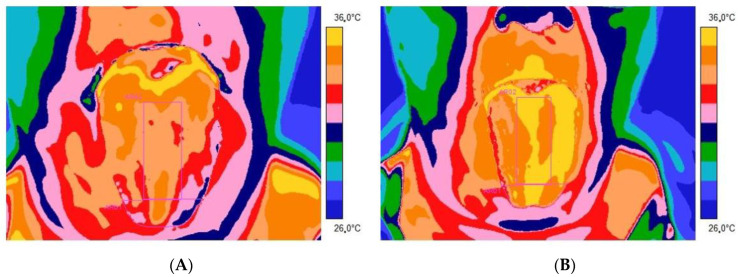
Thermal images of representative patients were obtained 2 (**A**) and 10 (**B**) minutes after rinsing the mouth with water at room temperature for 60 s. There are Regions of Interest marked on the tongue.

**Figure 2:**
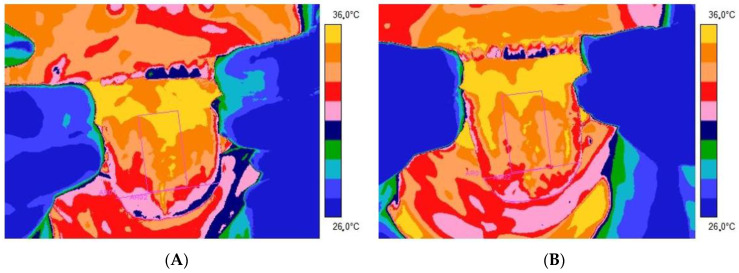
Thermal images of representative volunteers from the control group were obtained 2 (**A**) and 10 (**B**) minutes after rinsing the mouth with water at room temperature for 60 s. There are Regions of Interest marked on the tongue.

Figure 1 and Figure 2 present thermal images of representative patients and volunteers from the control group tongue obtained 2 (Figure 1A and Figure 2A) and 10 (Figure 1B and Figure 2B) minutes after rinsing the mouth with water at room temperature for 60 s.

From Table 1 and Table 2, as well as the thermal imaging presented, it is easy to see that the average temperature obtained from both the apex and the dorsum part of the tongue is higher in people in the healthy control group than in the patients of the research group presented. Moreover, the adjusted method relies on rinsing the mouth with water at room temperature for 60 s, differentiating the tongue temperature response between studied groups. It seems that the temperature indicated as healthy is significantly higher than in the case of patients with disorders. It is especially seen after 10 min from mouth rinsing.

For better insights into the problem, the statistical analysis has been conducted, and, besides average temperature, the temperature difference (defined as DT = average temperature 10 min − average temperature 2 min after rinsing of mouth) has been taken into consideration.

According to proper statistical analysis first, all data were checked for normality distribution by using the Shapiro–Wilk’s test. We found that the studied variables were not normally distributed and did not meet the homogeneity of the variance assumption, which was why they were subjected to the Mann–Whitney U test. Differences were statistically significant with *p* < 0.05.

It was obtained that there were no statistical differences between the studied parameters measured and calculated 2 min after rinsing the mouth.

However, further analysis showed statistics significant differences in the average temperature of studied tongue areas (TA and TD) measured 10 min after rinsing the mouth as well as in temperature difference, defined as DT = T_ave10_ (average temperature 10 min after rinsing of mouth) − T_ave2_ (average temperature 2 min after rinsing of mouth) between patients and the control group.

It seems that it is necessary to keep the return process to thermodynamic equilibrium after mouth and tongue cooling a little longer to see the differences in the dynamics of the thermoregulation process occurring between studied groups.

**Figure 3:**
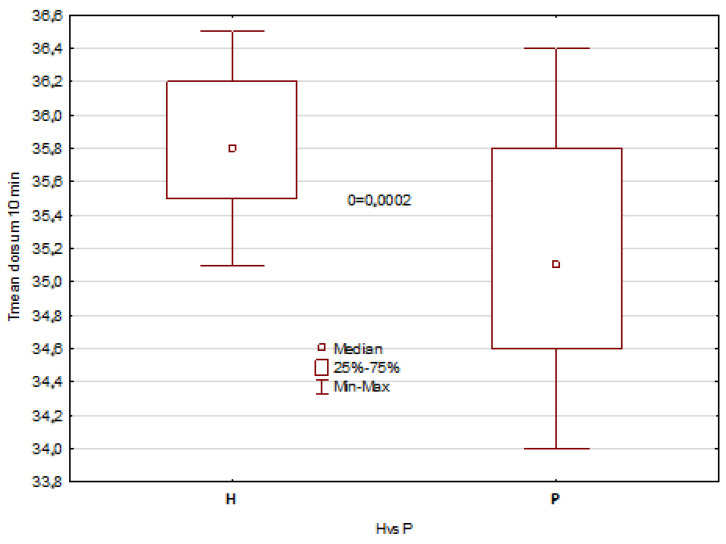
Boxes graph representing the differences between the average temperature of tongue dorsum (TD) in healthy and patients.

**Figure 4:**
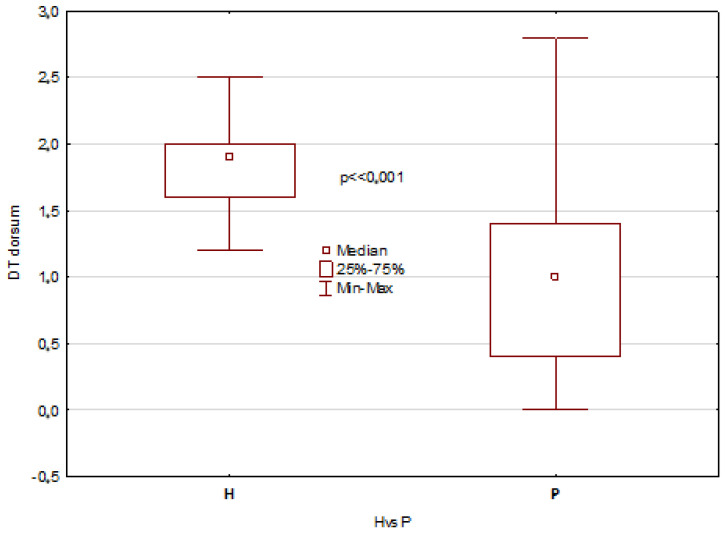
Boxes graph representing the differences in DT (defined as DT = average temperature 10 min − average temperature 2 min after rinsing of mouth) of tongue dorsum (TD) in healthy and patients.

**Figure 5:**
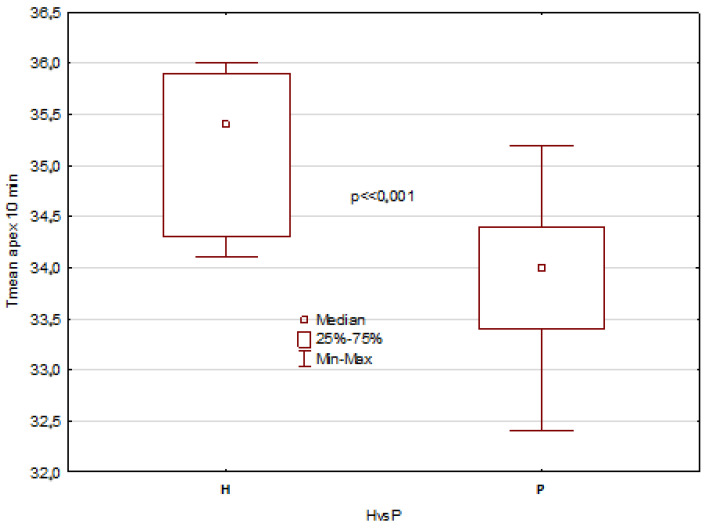
Boxes graph representing the differences between the average temperature of tongue apex (TA) in healthy and patients.

**Figure 6:**
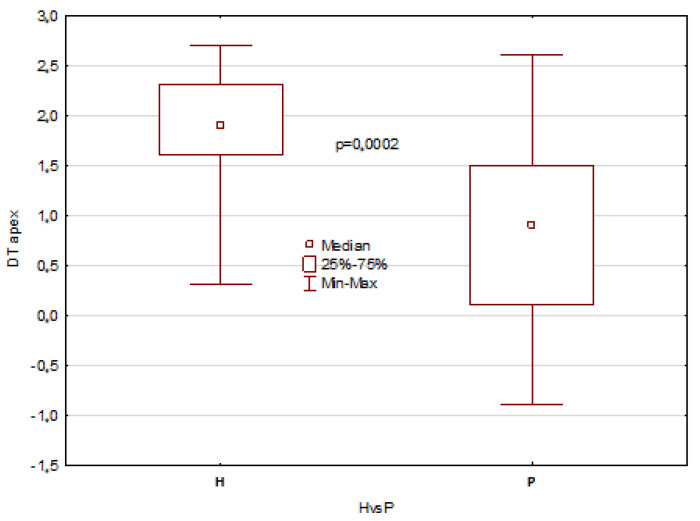
Boxes graph representing the differences in DT (defined as DT = average temperature 10 min − average temperature 2 min after rinsing of mouth) of tongue apex (TA) in healthy and patients.

The mentioned results describe all temperature parameters, which showed clearly that significant differences between healthy patients and patients with disorders are seen 10 min after mouth rinsing and in temperature difference DT, as shown in Figure 3, Figure 4, Figure 5 and Figure 6.

The obtained results revealed statistically significant differences in the tongue average temperature parameters between the two study groups. There were obtained median temperatures for TD of 35.8 °C and 35.1 °C for healthy patients and patients with disorders, respectively. And the differences were statistically significant with *p* = 0.0002. Similar statistical results and significant differences have been obtained for TA average temperature, and the obtained values were 35.5 °C and 34.0 °C for healthy patients and patients with disorders, respectively (*p* << 0.001). It should be also underlined that average temperature differences of TA area obtained between 10 and 2 min after rinsing were strongly significant, and the obtained values for healthy were about 1 °C higher than for unhealthy patients (Figure 3 and Figure 5). Moreover, the DT parameter defined as average temperature 10 min − average temperature 2 min after rinsing of mouth showed also very strong statistically significant difference (*p* << 0.05) between the healthy group and unhealthy patients, and the obtained values were in both cases nearly 1 °C (Figure 4 and Figure 6).

## 4. Discussion

In traditional Chinese medicine (TCM), the tongue has been the most important diagnostic tool for thousands of years, the role of which has often been questioned and discussed by scientists. In their opinion, this organ is connected to the internal organs through meridians and is, therefore, representative of pathological states of “cold” or “warm”. With the increasing knowledge in the field of molecular biology, a close relationship between tongue muscle mass and many systemic diseases has been documented. The tongue is a collective tissue of muscle fibers capable of reflecting a disease state in the body. In the literature, there is a clinical assessment of the tongue based on color, shape, moisture, and thickness, which is used for the diagnosis of various diseases [15,16,17,18,19,20,21,22].

Even though tongue diagnosis is convenient and non-invasive, it has been difficult to obtain objective and standardized tests. Changes in test conditions, such as energy sources, may significantly affect the result. Over the years, many research projects have tried to solve these problems, bearing in mind that the diagnosis must be based on the experience and knowledge of the doctor.

Human health is strongly correlated with body temperature, so the tongue, too, is within a narrowly defined range in healthy people. Under standardized conditions of the measuring environment, deviations in body temperature values may indicate pathological processes. Therefore, high hopes are placed on thermography as a scanning technique that brings the thermal map of temperature showing the differences on the surface [19].

Constant technological progress, both in the field of thermographic devices and the possibilities of subsequent computer image processing, influenced the ease and reliability of this method. Protocols for the standardization of examination repeatability and image evaluation via artificial intelligence opened up great opportunities for the wide application of non-invasive thermographic methodology in the medical and diagnostic field [19,20]. According to the Chinese school, the classic model of language segmentation due to shape, color, and size has many imperfections. With the development of information technology, language segmentation can be refined using a two-phase convolutional neural network that is not influenced by external factors [20].

It has been reported that the assessment of tongue temperature using IRT is used in the diagnosis of diabetes and anemia based on local secondary changes in temperature to changes in blood flow [9,10]. Moreover, thermal imaging is trying to be used as a screening tool to detect cancer and monitor disease progression. According to the literature, lowered temperature values for various diseases were observed on tongue thermograms. In a study by Xie and Zhang, the tongue thermograms of people with anemia had a lower temperature (34.14 °C). Baek et al. found a decrease in tongue temperature (32.70 °C) for cold and warm patterns in women with gynecological problems. However, in the case of type I diabetic tongue thermograms, a higher temperature was found (35.94 °C) than in other diseases [10,20,21].

Due to the good blood supply to the tongue, its surface temperature reflects the internal temperature carried by the bloodstream. Infrared thermography (IRT) provides an image of the temperature distribution by converting the intensity of thermal radiation emitted from the surface of any object to the temperature value at individual points of this surface. The IRT technique effectively monitors the surface temperature of the human body, which is affected by factors such as blood flow at the surface of the body and heat conduction in deeper blood vessels. Due to its convenience and non-invasiveness, the IRT technique has been used to study some of the physiological characteristics of tongue temperature, including the effect of age and regional differences. Korean researchers claimed that tongue temperature, as estimated via IRT, was related to age and gender and that there were regional temperature variations. They showed that a decrease in tongue temperature may be related to a decrease in the rate of blood perfusion in the tongues of the elderly. Studies have shown that the temperature at the base of the tongue was the highest and gradually decreased towards the center of the tongue, while the apex of the tongue had the lowest temperature [17].

As proposed in performed studies, the method of inner mouth cooling using room temperature (20–22 °C) water to cool down the tongue provides two processes—differentiating the temperature of the muscle and leading to an increase in blood supply in the tongue and the thermoregulation so the metabolism. The efficiency of those processes must be correlated with systemic disorders, i.e., metabolic, blood supply, or hormonal [23]. Cooling mouth tissues does not have an influence on the whole organism and does not disturb the organism’s metabolism, so it is as safe and non-invasive for patients as thermal imaging. However, importantly, it leads to different blood supply in the tongue, and the differences return to the thermal equilibrium of the very good blood-supplied tongue between unhealthy patients and healthy ones. The results draw our attention to the conclusion that the time of return of the temperature of the muscle seems to be most important. It may bring fast and completely non-contact information about patient systematic disorders. So, it seems that the thermovision diagnostic procedure, including thermal imaging of the tongue 10 min after rinsing the mouth with water at room temperature for 60 s, may be sufficient and bring important information that may show how to plan the surgery or other incriminating dentistry surgical procedures.

The authors of this study tried to demonstrate the usefulness of thermal imaging in patient screening, showing that patients with general diseases show a different reaction to temperature than healthy people. The limitation of this study was a relatively small, heterogeneous research group. Therefore, it is necessary to conduct further research, increasing the number of subjects and distinguishing subpopulations of patients with various systemic diseases.

## 5. Conclusions

The presented author’s method of the thermovision measurement of temperature distribution on the surface of temperature the tongue can be a prognostic tool for diagnosing general diseases manifested by fluctuations in internal temperature, i.e., dynamic changes in tongue temperature, due to the cooling difference between healthy and patients. We found that the average temperature as well as the temperature difference in the TA and TD areas calculated between 10 and 2 min after mouth rinsing were strongly significant, and the values for healthy were about 1 °C higher than for patients in both cases.

The thermographic examination of the tongue can become a quick and effective diagnostic test or facilitate the quantitative assessment of applied therapies by demonstrating temperature differences in its various sectors. It is necessary to confirm the results of our pilot on a wider group of patients. This study presents interesting possibilities for using IRT as a harmless screening tool with great potential in the prevention and diagnosis of general diseases.

## Figures and Tables

**Table 1:** Values of the mean temperature of the dorsum and apex of the tongue after 2 min and 10 min and differences in 33 patients of the study group.

No	T Mean Tongue Dorsum after 2 min	T Mean Tongue Dorsum after 10 min	DT Tongue Dorsum	T Mean Tongue Apex after 2 min	T Mean Tongue Apex after 10 min	DT Tongue Apex
1	35.7	35.8	0.1	33.6	34.4	0.8
2	35.7	35.8	0.1	33.7	34.4	0.7
3	34.9	35.3	0.4	34.4	33.8	−0.6
4	35.1	35.3	0.2	34	35.2	1.2
5	34.3	35.4	1.1	32.2	34.4	2.2
6	35.3	35.8	0.5	34.4	34	−0.4
7	34.8	35.8	1	33.4	33.1	−0.3
8	35.7	35.7	0	35.3	34.4	−0.9
9	35	36	1	33.8	35.2	1.4
10	33.1	35	1.9	33.5	34.4	0.9
11	33.6	36.4	2.8	32.6	35.2	2.6
12	33.7	35.1	1.4	31.6	33.7	2.1
13	35	36	1	33.8	35.2	1.4
14	33.6	36.4	2.8	32.6	35.2	2.6
15	33.7	35.1	1.4	31.6	33.7	2.1
16	33.6	34.1	0.5	31.2	32.5	1.3
17	32.9	34.7	1.8	32.6	34.5	1.9
18	34.2	34.6	0.4	32.6	33.3	0.7
19	34.2	34.6	0.4	33.2	33.7	0.5
20	34	35.5	1.5	32.6	32.4	−0.2
21	33.3	34.6	1.3	33.7	33.4	−0.3
22	33.6	34.4	0.8	33.1	34	0.9
23	32.2	34.2	2	30.5	32.9	2.4
24	34.1	35.1	1	33.1	34.1	1
25	32.7	34.5	1.8	32.7	34.2	1.5
26	34.6	35.5	0.9	33.2	34.1	0.9
27	35	35.8	0.8	33.6	34.1	0.5
28	33.9	34.1	0.2	33.7	33.6	−0.1
29	33.8	34.8	1	33.1	34.6	1.5
30	33.7	34.5	0.8	32.5	33.6	1.1
31	33.5	34	0.5	32.9	33	0.1
32	34.3	34.3	0	33	32.5	−0.5
33	33.7	35.2	1.5	31/9	33.3	1.4

**Table 2:** Values of the mean temperature of the dorsum and apex of the tongue after 2 min and 10 min and differences in 33 patients of the control group.

No	T Mean Tongue Dorsum after 2 min	T Mean Tongue Dorsum after 10 min	DT Tongue Dorsum	T Mean Tongue Apex after 2 min	T Mean Tongue Apex after 10 min	DT Tongue Apex
1	33.9	35.8	1.9	33.1	34.7	1.6
2	33.8	36.3	2.5	33.2	35.9	2.7
3	34.6	36.2	1.6	33.7	36	2.3
4	33.6	35.5	1.9	33.7	35.6	1.9
5	33.3	35.1	1.8	32.4	34.3	1.9
6	34.3	35.5	1.2	33.9	34.2	0.3
7	34.5	35.8	1.3	33.6	34.1	0.5
8	34.3	36.5	2.2	33.1	35.6	2.5
9	33.9	35.8	1.9	33.1	34.7	1.6
10	33.8	36.3	2.5	33.2	35.9	2.7
11	34.6	36.2	1.6	33.7	36	2.3
12	33.6	35.5	1.9	33.7	35.6	1.9
13	33.3	35.1	1.8	32.4	34.3	1.9
14	34.3	35.5	1.2	33.9	34.2	0.3
15	34.5	35.8	1.3	33.6	34.1	0.5
16	33.9	35.8	1.9	33.1	34.7	1.6
17	33.8	36.3	2.5	33.2	35.9	2.7
18	34.6	36.2	1.6	33.7	36	2.3
19	33.6	35.5	1.9	33.7	35.6	1.9
20	33.9	35.8	1.9	33.1	34.7	1.6
21	33.3	35.1	1.8	32.4	34.3	1.9
22	34.3	35.5	1.2	33.9	34.2	0.3
23	34.5	35.8	1.3	33.6	34.1	0.5
24	33.9	35.8	1.9	33.1	34.7	1.6
25	33.6	35.5	1.9	33.7	35.6	1.9
26	33.8	36.3	2.5	33.2	35.9	2.7
27	33.9	35.8	1.9	33.1	34.7	1.6
28	34.6	36.2	1.6	33.7	36	2.3
29	33.6	35.5	1.9	33.7	35.6	1.9
30	33.3	35.1	1.8	32.4	34.3	1.9
31	34.3	35.5	1.2	33.9	34.2	0.3
32	34.5	35.8	1.3	33.6	34.1	0.5
33	33.9	35.8	1.9	33.1	34.7	1.6

## Data Availability

Not applicable.

## References

[1] Modrzejewska M., Parafiniuk M. (2018). Application of thermography in medicine—Literature review. Pomeranian J. Life Sci..

[2] Jung A., Kalicki B., Żuber J., Ring E.F.J. (2013). Infrared thermal imaging as noninvasive method of body temperature measurement in hospitalized and nonhospitalized children. Overv. Electrotechnical..

[3] Ring EF J., McEvoy H., Jung A., Zuber J., Machin G. (2010). new standards for devices used for the measurement of human body temperature. J. Med. Eng. Technol..

[4] Bauer J., Dereń E. (2014). Standardization of infrared thermal imaging in medicine and physiotherapy. Acta Bio-Opt. Et Inform. Medica.

[5] Ring EF J., Jung A., Kalicki B., Zuber J., Rustecka A., Vardasca R. (2013). new standards for fever screening with thermal imaging system. J. Mech. Med. Biol..

[6] Nicolas-Rodriguez E., Garcia-Martinez A., Molino-Pagan D., Marin-Martinez L., Pons-Fuster E., López-Jornet P. (2022). Thermography as a non-ionizing quantitative tool for diagnosing burning mouth syndrome: Case-control study. Int. J. Environ. Res. Public. Health.

[7] Gorczewska I., Szurko A., Kiełboń A., Stanek A., Cholewka A. (2022). Determination of internal temperature by measuring the temperature of the body surface due to environmental physical factors-first study of fever screening in the COVID pandemic. Int. J. Environ. Res. Public Health.

[8] Wziątek-Kuczmik D., Niedzielska I., Mrowiec A., Bałamut K., Handzel M., Szurko A. (2022). Is thermal imaging a helpful tool in diagnosis of asymptomatic odontogenic infection foci—A pilot study. Int. J. Environ. Res. Public Health.

[9] Thirunavukkarasu U., Umapathy S., Krishnan P.T., Janardanan K. (2020). Human Tongue Thermography Could Be a Prognostic Tool for Prescreening the Type II Diabetes Mellitus. Evid. Based Complement. Alternat Med..

[10] Xie H., Zhang Y. (2018). Relationship between dynamic infrared thermal images and blood perfusion rate of the tongue in anemia patients. Infrared Phys. Technol..

[11] Kaszuba N., Kasprzyk-Kucewicz T., Szurko A., Wziatek-Kuczmik D., Stanek A., Morawiec T., Cholewka A. (2021). How to use thermal imaging in selected surgical dental procedures?. Thermol. Int..

[12] Aboushady M.A., Talaat W., Hamdoon Z., M.Elshazly T., Ragy N., Bourauel C., Talaat S. (2021). Thermography as a non-ionizing quantitative tool for diagnosing periapical inflammatory lesions. BMC Oral. Health.

[13] Christensen J., Matzen L.H., Vaeth M., Schou S., Wenzel A. (2012). Thermography as a quantitative imaging method for assessing postoperative inflammation. Dentomaxillofac Radiol..

[14] Christensen J., Matzen L., Vaeth M., Schou S., Wenzel A. (2021). The Thermal influence of oral rehabilitation on the cranio- cervico -mandibular complex: A thermographic analysis. Int. J. Environ. Res. Public. Health..

[15] Bagaria R., Wadhwani S. (2017). Tongue diagnosis by image segmentation. Int. J. Adv. Innov. Res..

[16] Jung C.J., Jeon Y.J., Kim J.Y., Kim K.H. (2012). Review on the current trends in tongue diagnosis systems. Integr. Med. Res..

[17] Schnorrenberger C., Schnorrenberger B. (2012). Diagnosing with Language. Practical Tips on Treatment with Acupuncture, Herbs and Diet.

[18] Maciocia G. (1999). Tongue Diagnosis in Chinese Medicine.

[19] Kesztyüs D., Brucher S., Kesztyüs T. (2022). Use of infrared thermography in medical diagnostics: A scoping review protocol. BMJ Open.

[20] Baek S.-W., Lee J.-M., Park Y.-J. (2018). Relationship between Tongue Temperature Estimated by Infrared Thermography, Tongue Color, and Cold-Heat Pathological Patterns: A Retrospective Chart Review Study. Evid. Based Complement. Alternat Med..

[21] Zhang H., Jiang R., Yang T., Gao J., Wang Y., Zhang J. (2022). Study on TCM tongue image segmentation model based on convolutional neural network fused with superpixel. Evid. Based Complement. Alternat Med..

[22] Selvarani A., Suresh G.R. (2019). Infrared thermal imaging for diabetes detection and measurement. J. Med. Syst..

[23] Podbielska H., Skrzek A. (2012). The Use of Low Temperatures in Biomedicine.

